# Shear bond strength and adhesive remnant index of orthodontic brackets bonded to enamel using adhesive systems mixed with TiO_2_ nanoparticles

**DOI:** 10.1590/2177-6709.23.4.43.e1-7.onl

**Published:** 2018

**Authors:** Mohammad Behnaz, Kazem Dalaie, Hoori Mirmohammadsadeghi, Hamed Salehi, Vahid Rakhshan, Farzin Aslani

**Affiliations:** 1 Shahid Beheshti University of Medical Sciences, Research Institute of Dental Sciences, Dentofacial Deformities Research Center (Tehran, Iran). Shahid Beheshti University of Medical Sciences Shahid Beheshti University of Medical Sciences Research Institute of Dental Sciences Dentofacial Deformities Research Center Tehran Iran; 2 Shahid Beheshti University of Medical Sciences, School of Dentistry, Department of Orthodontic (Tehran, Iran). Shahid Beheshti University of Medical Sciences Shahid Beheshti University of Medical Sciences School of Dentistry Department of Orthodontic Tehran Iran; 3 Private practice (Tehran, Iran). Tehran Iran

**Keywords:** Titanium dioxide, Nanoparticles, Orthodontic brackets, Shear bond strength

## Abstract

**Introduction:**

It is recently suggested that titanium dioxide (TiO_2_) nanoparticles can be added to bracket luting agents in order to reduce bacterial activity and protect the enamel. However, it is not known if this addition can affect the shear bond strength (SBS) below clinically acceptable levels. Therefore, this study examined this matter within a comprehensive setup.

**Methods:**

This in vitro experimental study was conducted on 120 extracted human premolars randomly divided into four groups (n=30): in groups 1 and 2, Transbond XT light-cured composite with or without TiO_2_ was applied on bracket base; in groups 3 and 4, Resilience light-cured composite with or without TiO_2_ was used. Brackets were bonded to teeth. Specimens in each group (n=30) were divided into three subgroups of 10 each; then incubated at 37°C for one day, one month, or three months. The SBS and adhesive remnant index (ARI) were calculated and compared statistically within groups.

**Results:**

The SBS was not significantly different at one day, one month or three months (*p*>0.05) but composites without TiO_2_ had a significantly higher mean SBS than composites containing TiO_2_ (*p*<0.001). The SBS of Transbond XT was significantly higher than that of Resilience (*p*<0.001). No significant differences were noted in ARI scores based on the type of composite or addition of TiO_2_ (*p*>0.05).

**Conclusions:**

Addition of TiO_2_ nanoparticles to Transbond XT decreased its SBS to the level of SBS of Resilience without TiO_2_; thus, TiO_2_ nanoparticles may be added to Transbond XT composite for use in the clinical setting.

## RESUMO

**Introdução:**

recentemente, sugeriu-se que nanopartículas de dióxido de titânio (TiO_2_) poderiam ser adicionadas ao cimento adesivo para reduzir a atividade bacteriana e proteger o esmalte. Entretanto, não se sabe se esse acréscimo pode reduzir a resistência adesiva ao cisalhamento (RAC) a níveis inferiores aos clinicamente aceitáveis. Assim, o presente estudo examinou essa questão dentro de um contexto abrangente.

**Métodos:**

esse estudo experimental *in vitro* foi realizado em 120 pré-molares humanos, aleatoriamente divididos em quatro grupos (n=30). Nos grupos 1 e 2, o adesivo fotopolimerizável Transbond XT com e sem TiO_2_ foi aplicado na base do braquete. Nos grupos 3 e 4, utilizou-se o adesivo fotopolimerizável Resilience com e sem TiO_2_. Os braquetes foram colados aos dentes e as amostras de cada grupo (n=30) foram divididas em três subgrupos de dez amostras cada, as quais foram incubadas a 37°C por, respectivamente, um dia, um mês e três meses. A RAC e o índice de adesivo remanescente (IAR) foram calculados e estatisticamente comparados entre os grupos.

**Resultados:**

a RAC não apresentou diferença significativa após um dia, um mês ou três meses (*p* > 0,05), mas os adesivos sem TiO_2_ apresentaram uma RAC média significativamente mais elevada do que os adesivos que continham TiO_2_ (*p*< 0,001). A RAC do Transbond XT foi significativamente mais elevada do que a do Resilience (*p*< 0,001). Não foram observadas diferenças significativas nos IARs, seja para o tipo de adesivo ou para a adição de TiO_2_ (*p*> 0,05).

**Conclusões:**

a adição de nanopartículas de TiO_2_ ao Transbond XT reduziu sua RAC a níveis semelhantes aos da RAC do Resilience TiO_2_. Assim, as nanopartículas de TiO_2_ podem ser acrescentadas ao adesivo Transbond XT para a aplicação clínica.

## INTRODUCTION

Orthodontic brackets should endure masticatory forces, by proper adhesion to the enamel, which is reflected in vitro by shear bond strength (SBS).^1^^,^^2^ Loosely bonded brackets might dislodge or break,^3^ exerting extra expenses to the clinician and patient in terms of financial, time, and enamel damage (caused by resin removal methods before bonding of new brackets).^3^^-^^6^ Therefore, attempts have been made to improve the characteristics of composite resins used to bond orthodontic brackets. Currently micro-filled, micro-hybrid, and flowable composites are mainly used for orthodontic bracket bonding. However, commonly used orthodontic composites often have high polymerization shrinkage, low compressive and tensile strengths, low fracture strength and poor marginal seal.^7^ Nano-composites are the latest technology in the field of restorative composites. Due to the nanometer scale size of their filler particles (0.1 to 100nm), they have very high filler content, which improves their polymerization shrinkage, compressive and tensile strengths, fracture strength and marginal seal, compared to other composites.^8^

Despite all the material improvements, orthodontic brackets still accumulate bacterial plaque. Microbial toxins, enzymes, and acidic byproducts can result in formation of white spots or caries, gingival inflammation, periodontal problems, and increased metal ion release.^9^^-^^16^ Orthodontic treatment might cause enamel demineralization or formation of white spot lesions around orthodontic brackets in many orthodontic patients.^17^^-^^22^ This is especially important in Orthodontics when many patients cannot effectively maintain a perfect oral hygiene.^14^ Various methods and materials including fluoride or antibacterial agents have been proposed to reduce such side effects.^15^^,^^18^^,^^22^^-^^25^ Nanotechnology is employed in dental materials to improve mechanical properties and develop antimicrobial influences.^21^^,^^25^^,^^26^ Some composite fillers such as TiO_2_ have antibacterial properties, and their addition to composites may promote dental health.^22^ Titanium dioxide is an inorganic filler, which is non-toxic and biocompatible, and has optimal antibacterial, optical and electrical properties.^27^ Nanoparticles of TiO_2_ have proper mechanical, photocatalytic, and antimicrobial characteristics; also they are available in different crystalline formats and sizes, and are believed to be proper for addition into dental materials.^14^^,^^26^ Proper antibacterial effects of TiO_2_ nanoparticles have been previously confirmed.^14^^,^^15^^,^^22^^,^^28^^,^^29^ Therefore, its incorporation into bracket adhesives is suggested. 

However, it is not known whether the addition of such nanoparticles to the luting agent might or might not disrupt the bond strength, since the literature on this matter is scarce and controversial. To our knowledge, there are only three studies in this regard. Poosti et al^22^ compared the SBS of two groups of brackets bonded using a light-cure composite with and without TiO_2_ nanoparticles, and found no significant SBS differences after only 1 day of incubation.^22^ On the other hand, Reddy et al^14^ compared SBS values obtained using luting agents with or without nanoparticles of TiO_2_ (and without any aging or incubation), and showed a significant 30% decrease in the SBS after TiO_2_ incorporation. Felemban and Ebrahim^1^ reported in 2017 that addition of ZrO_2_-TiO_2_ nanoparticles to orthodontic adhesive might improve compressive, tensile, and shear bond strengths of orthodontic brackets. Since studies in this regard are few, this research was conducted. Its aim was to assess the effect of addition of TiO_2_ nanoparticles to orthodontic composites on the SBS of orthodontic brackets to enamel and the adhesive remnant index (ARI) scores in 120 human premolars.

## MATERIAL AND METHODS 

### Preparation of the samples

This in vitro, experimental study was conducted on 120 freshly extracted sound human premolars, which had been extracted for orthodontic purposes. The teeth were stored in 0.5% chloramine T solution at room temperature. The inclusion criteria were freshly extracted sound human premolars, which had not been subjected to any chemical treatment (such as bleaching or exposure to alcohol) prior to extraction. The exclusion criteria were presence of defects, cracks or caries. 

First, in a pilot study, the SBS of anatase and rutile mineral forms of TiO_2_ nanoparticles was measured, and anatase TiO_2_ nanoparticles were selected for use in this experiment due to having higher SBS. 

**Figure 1:**
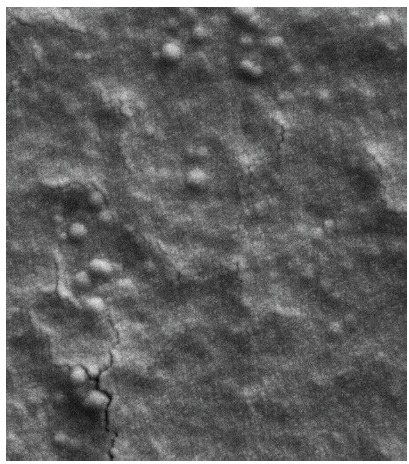
An example of Transbond XT + TiO_2_.

**Figure 2:**
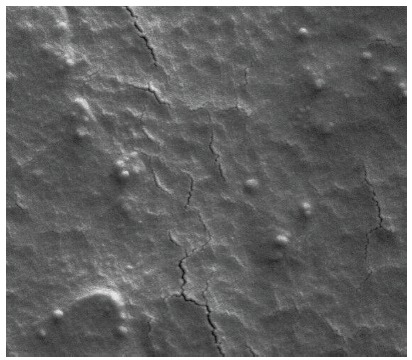
An example of Resilience + TiO_2_.

Anatase TiO_2_ nanoparticles in 0.1 wt% concentration were added to composites in a dark room after being weighed by a digital scale and mixed by a stirrer to produce a homogenous blend. To ensure that a homogenous blend was obtained, the mixture was inspected under an electron microscope (KYKY-EM3200, USA, Figs 1 and 2). 

The teeth were vertically mounted in auto-polymerizing acrylic blocks. The buccal surface of tooth crown was polished using fluoride-free pumice paste, and it was rinsed and dried. The buccal enamel was etched with 37% phosphoric acid gel for 20 seconds, rinsed from a 10-15cm distance for 40 seconds and was completely dried with oil- and moisture-free air blow to obtain the chalky white appearance of enamel. 

### Groups 

Eventually, the samples were randomly divided into four groups as follows:

» Group one (Transbond XT): Transbond XT primer (3M Unitek, Monrovia, CA, USA) was applied as a thin coat on the etched enamel, spread on the surface by gentle air spray from a 15cm distance, and cured for 10 seconds. Transbond XT composite (3M Unitek) was applied on bracket base (American Orthodontics, Sheboygan, USA). The bracket was placed on the middle third of the buccal enamel surface. Adequate pressure was applied by an explorer to the slot, in order to adapt the bracket to the tooth surface.
» Group two (Transbond XT plus TiO_2_): Transbond XT primer was applied as a thin coat on the etched enamel and cured for 10 seconds. Transbond XT plus TiO_2_ composite was applied on the bracket base, and the bracket was adapted to the enamel surface as in group one.
» Group three (Resilience): Resilience primer (Ortho Technology, Florida, USA) was applied as a thin coat on the etched enamel and cured for 10 seconds. Resilience composite (Ortho Technology, Lutz, Florida, USA) was placed on the bracket base, and the bracket was adapted to the enamel surface as in group one.
» Group four (Resilience plus TiO_2_): Resilience primer was applied as a thin coat on the etched enamel and cured for 10 seconds. Resilience composite plus TiO_2_ nanoparticles was placed on the bracket base and the bracket was adapted to the enamel surface as in group one.

Excess composite in all four groups was removed by the sharp tip of a scaler; all samples were light-cured for 10 seconds from the mesial, 10 seconds from the distal, 10 seconds from the gingival and 10 seconds from the occlusal surface using a light curing unit (Woodpecker Guilin, Guangxi, China) with a light intensity of 1000 mW/cm^2^. Also, the light-curing unit was calibrated by a radiometer every 10 minutes, to ensure equal intensity of light for all samples. 

### Evaluation of shear bond strength

Afterwards, the teeth were placed in deionized distilled water and incubated at 37°C to allow water sorption. At the designated time points (one day, one month, and three months), the teeth were placed on the jig of an Instron machine (Janke & Kuknek, IKA-Laborte Chnik, Germany). The stainless steel blade of the Instron machine had 4.0 mm length and applied the load to the bracket at a crosshead speed of 1 mm/minute. The SBS was calculated in MegaPascal (MPa) unit by dividing the shear load by surface area of the bracket base.

### Assessment of Adhesive Remnant Index

After debonding, the ARI score was calculated based on the following scoring system under a 10× stereomicroscope (Olympus, Japan):

» Score zero: Indicated absence of composite remnants on the enamel surface.
» Score one: Less than 50% of composite remaining on the enamel surface.
» Score two: More than 50% of composite remaining on the enamel surface.
» Score three: The entire composite remained on the enamel surface with a clear impression of the bracket base on the remaining composite.

### Statistical analysis

The effects of time, type of composite and presence/absence of TiO_2_ nanoparticles on the SBS of brackets to enamel were analyzed using three-way analysis of variance (ANOVA). Also, comparisons of the groups in terms of ARI scores were made using the Mann-Whitney test. Changes in ARI scores over time (based on the duration of incubation of samples) were analyzed using the Kruskal-Wallis test of SPSS software (version 20, IBM, Armonk, NY, USA). Level of significance was predetermined as ≤ 0.05.

## RESULTS 

The mean and standard deviation (SD) of SBS based on the time of incubation, type of composite and presence/absence of TiO_2_ nanoparticles in the composites are presented in Table 1. The highest SBS was found in Transbond XT composite (145.73±3.87 MPa) followed by Resilience (125.59±3.37 MPa) without TiO_2_ nanoparticles. The lowest SBS was noted in Resilience plus TiO_2_ (77.75±2.33 MPa) followed by Transbond XT plus TiO_2_ (123.92±3.17 MPa) groups. Normal distribution of SBS data was ensured by the Kolmogorov-Smirnov test. Since the data were normally distributed and considering the equality of variances confirmed by Levene’s test, three-way ANOVA was used to compare the SBS values in the four groups. The three-way ANOVA revealed no significant difference in SBS of the groups over time (*p*=0.94); however, the mean SBS was significantly higher in the groups of pure composites without TiO_2_ nanoparticles compared to the value in composites containing TiO_2_ (*p*<0.001). Also, the mean SBS of Transbond XT composite was significantly higher than that of Resilience composite (*p*<0.001) and the interaction effect of type of composite and presence/absence of TiO_2_ on SBS was statistically significant (*p*<0.001). In Transbond XT composite without TiO_2_, the mean SBS value was about 20 units higher than that in Transbond XT containing TiO_2_. This difference in Resilience groups was 40 units. The other interaction effects were not significant (*p*>0.05 for all comparisons). 

**Table 1:** Statistics of shear bond strength (MPa) at different time points in the four groups.

Brand	TiO_2_	Aging (day)	Mean	SD	SE	95% CI	
Transbond X	No	1	147.44	6.28	1.99	142.95	151.93
		30	146.08	3.95	1.25	143.25	148.91
		90	143.66	9.41	2.98	136.93	150.39
	Yes	1	126.43	5.93	1.88	122.19	130.67
		30	120.99	5.84	1.85	116.81	125.17
		90	124.33	5.09	1.61	120.69	127.97
Resilience	No	1	125.62	6.65	2.10	120.86	130.38
		30	124.12	6.83	2.16	119.23	129.01
		90	127.05	4.26	1.35	124.00	130.10
	Yes	1	74.25	5.51	1.74	70.31	78.19
		30	78.66	3.62	1.14	76.07	81.25
		90	80.35	2.58	0.82	78.50	82.20

SD = standard deviation; SE = standard error; CI = confidence interval.

Table 2 shows the mean ARI scores in the four groups. According to the results of Mann-Whitney U test, no significant differences were noted in terms of ARI scores based on the type of composite used or presence/absence of TiO_2_ nanoparticles (*p*=0.43). The ARI scores did not change significantly over time according to the results of the Kruskal-Wallis test (*p*=0.19). 

**Table 2:** The mean ARI scores in the four groups.

Type of composite	Time	Mean	Score
Transbond XT	Three months	70%	2
	One month	100%	3
	One day	63%	2
Transbond XT + TiO_2_	Three months	55%	2
	One month	100%	3
	One day	77%	2
Resilience	Three months	100%	3
	One month	60.7%	2
	One day	95%	2
Resilience + TiO_2_	Three months	67%	2
	One month	80%	2
	One day	90%	2

## DISCUSSION 

An acceptable bracket bonding system must be able to resist destructive forces applied by orthodontic wires as well as the loads applied in the oral cavity.^30^^,^^31^ The present results showed that addition of TiO_2_ nanoparticles to orthodontic composites significantly decreased the mean SBS of both Transbond XT and Resilience composites. Also, the mean SBS did not significantly change over time. The mean SBS was significantly higher in composites without TiO_2_ compared to composites containing TiO_2_. In contrast to the findings of the current study, Felemban and Ebrahim^1^ reported that adding ZrO_2_-TiO_2_ nanoparticles might improve shear bond strength (together with tensile and compressive strengths). Furthermore, Poosti et al^22^ assessed the SBS of Transbond XT with and without addition of 1% TiO_2_ nanoparticles (less than 50nm in size) and found no significant difference in SBS of this composite with and without TiO_2_ at 24 hours.^22^ However, Reddy et al^14^ reported a significant 30% decrease in the SBS obtained using composites containing TiO_2_. A study on the addition of copper nanoparticles to orthodontic luting agents reported an increase in bond strength after nanoparticle addition.^23^ Blöcher et al^32^ evaluated the effect of addition of nano and microparticles of silver to orthodontic adhesive, and reported no significant change in SBS. Akhavan et al^33^ evaluated the effect of addition of silver nanoparticles/hydroxyapatite to Transbond XT orthodontic adhesive on SBS to enamel and found that addition of 1% to 5% silver nanoparticles/hydroxyapatite increased the SBS of adhesive, while addition of 10% silver nanoparticles/hydroxyapatite had no favorable effect on bond strength, compared to the control group.^33^ These differences can be attributed to various methodological variations, for instance: small sample sizes were small and might disallow identification of differences; moreover, particle sizes were not standardized across studies. It is possible that particles larger than a certain threshold might interfere with adhesive bonds more considerably while smaller particles might not. Additionally, different durations of aging procedures might affect results. Furthermore, different results pertaining to different types and brands of adhesives are not fully generalizable to other types and brands. Hence, their standardization would allow a better comparison of the effect of particle addition.^34^^,^^35^

In bracket bonding, in contrast to restorative treatments, very high bond strength is not always favorable, since the enamel surface would be damaged at the time of bracket debonding.^6^ A minimum SBS of about 6 to 10MPa might suffice to hold orthodontic brackets in place.^2^^,^^8^^,^^35^^-^^38^ Increasing the SBS to 13 MPa might increase the likelihood of cohesive failures and damage to ceramic restorations.^39^

Depending on brands in use, SBS varied greatly, as addition of TiO_2_ nanoparticles to Transbond XT composite decreased its bond strength to the level of SBS of Resilience composite without TiO_2_ in this study. Thus, certain brands of adhesives might provide higher bond strengths when needed. Uysal et al^8^ reported that Transbond XT yielded the highest SBS (12.6±4.48 MPa) followed by nano-composite (8.33±5.16 MPa) and nano-ionomer (6.14±2.12 MPa). 

Aging can weaken composite matrix by mechanisms such as swelling it, depleting its free radicals by water sorption or thermal stresses, and hydrolytic degradation of the silane film over fillers.^37^^,^^40^^-^^43^ However, this study did not show any significant differences between 1, 30, or 90 days of aging. It is possible that TiO_2_ nanoparticles might have improved resin structure and have reduced the deteriorating effect of aging. There was no study on the effect of aging on SBS of TiO_2_-incorporated resins, and future studies should evaluate this.

After bracket debonding, removal of resin from enamel side might be clinically favorable, as it might reduce damage caused by bracket debonding procedures.^36^^,^^37^ To assess the bracket debonding interface, ARI score is often calculated.^8^ Comparison of ARI scores based on the type of composite and presence/absence of TiO_2_ showed no significant difference in this regard. The ARI scores did not change significantly over time. Uysal et al^8^ reported no significant difference in ARI scores among Transbond XT composite, Filtek Supreme Plus Universal nano-composite and Ketac^TM^ N100 light-curing nano-ionomer. Similarly, Akhavan et al^33^ found no significant difference in ARI scores among 1%, 5% and 10% silver nanoparticles/hydroxyapatite plus Transbond XT primer. In their study, addition of silver nanoparticles/hydroxyapatite to Transbond XT orthodontic adhesive caused no significant difference in ARI scores of the groups.^33^ On the other hand, according to Nagar et al,^44^ ARI scores were not significantly different between the two groups of Transbond XT and nano-ceramic composites, which was in agreement with the current results. 

This study was limited by some factors. A sample size calculated based on pilot studies could favor the reliability. Moreover, in vitro experiments of bond strength cannot be generalized to clinical situations where different forces are exerted from various directions over brackets.^38^ In addition, results pertaining to a specific brand of some material cannot be generalized to other brands or formulas.^38^ Some differences exist among tensile, shear and torsional loads; however, shear loads are among the most common and most destructive forces in the oral cavity.^30^^,^^31^ Although these are standard tests, they cannot simulate the actual loads applied in the oral environment because the speed of jaw movements during mastication is in the range of 81-100mm/second or 4860-6000 mm/minute with a frequency of 1.03-1.2 Hz, which is different from the selected crosshead speeds for SBS testing.^45^

## CONCLUSIONS 

The addition of TiO_2_ nanoparticles might reduce SBS, but the adhesion might still be at an acceptable level. Transbond XT and Resilience without TiO_2_ nanoparticles yielded the highest SBS values, respectively. However, addition of TiO_2_ nanoparticles to Transbond XT decreased its SBS to the level of SBS of Resilience without TiO_2_. Thus, TiO_2_ nanoparticles may be added to Transbond XT composite. 
